# Exploring attitudes and variation by sociodemographic factors in consent provided for financial data linkage in an experimental birth cohort study

**DOI:** 10.1186/s12889-024-18226-1

**Published:** 2024-03-05

**Authors:** Sian Reece, Josie Dickerson, Kate E. Pickett

**Affiliations:** 1 Hull York Medical School, York, North Yorkshire UK; 2 Bradford Institute for Health Research, Bradford Royal Infirmary, Bradford, West Yorkshire UK; 3 Department of Health Sciences, University of York, Heslington Road, York, North Yorkshire UK

**Keywords:** Cohorts, Born in Bradford, data linkage, ethnicity, mental health, Poverty, Health inequalities, Social inequalities, Ethnicity

## Abstract

**Background:**

Improving our understanding of household incomes and what constitutes financial insecurity can help us to better understand how financial insecurity is experienced and how this can change over time within and between individuals and populations. However, financial circumstances are often perceived as sensitive and stigmatising, particularly within some ethnic minority groups. This research aims to explore attitudes and variation by sociodemographic factors in consent provided for financial data linkage in an experimental birth cohort study, in order to obtain validated income and benefits data and to better understand the impact of community interventions on the financial security of its participants and their families.

**Methods:**

This research utilises an observational study design to explore consent rates, attitudes and variation in sociodemographic factors between participants of an experimental birth cohort in a deprived and ethnically diverse setting who consent and do not consent to financial data linkage.

**Results:**

Overall, participants were equally likely to consent and decline consent for financial data linkage. Measures of socioeconomic insecurity were associated with being more likely to provide consent for financial data linkage. Participants who were not employed (OR 1.49 95% CI 0.93, 2.40) and were more financially insecure (OR 1.85 95% CI 1.14, 3.93) were more likely to provide consent for financial data linkage. Where the participant’s first language was a language other than English, participants were also less likely to provide consent for data linkage (OR 0.65 95% CI 0.39, 0.98). The choice of consent for financial data linkage was not associated with: ethnicity; relationship factors; employment status of the participant’s partner; person present at time of recruitment; and measures of health, such as general health, mental health, wellbeing and health-related quality of life.

**Conclusions:**

This research sets out an approach to obtaining validated income and benefits data, as a proxy measure for financial security, within an experimental birth cohort study in a deprived and ethnically diverse setting. It achieves good consent rates and demonstrates greater input from those who report greater potential need for financial support. Further research should be conducted to further understand the interplay of language spoken in this context.

**Supplementary Information:**

The online version contains supplementary material available at 10.1186/s12889-024-18226-1.

## Background

Improving our understanding of household incomes and what constitutes financial insecurity for families can help us to better understand how financial insecurity is experienced and how this can change over time within and between individuals and populations. Having an accurate understanding of financial insecurity can also help researchers, commissioners and policy makers to have a better understanding of what works to improve the financial security of individuals and communities and the impact this has on health and wellbeing and health inequalities. Validated financial outcome data can also facilitate and improve the accuracy of health economic analyses.

### Financial security data

Information regarding financial security can be obtained from a variety of data sources. Self-reported measures of income and financial security are useful in improving understanding of a person’s subjective assessment and experience of financial insecurity. However, there are significant challenges inherent in obtaining valid information about financial circumstances and security. Financial data can be seen as sensitive and stigmatising. Financial security may be over or under-estimated depending on the context and may give rise to social desirability bias. For populations vulnerable to financial insecurity arising from short-term employment, contractual jobs, and for independent contractors, measures of financial income may be subject to recall bias. Gaining an in depth understanding of the financial security of participants and their families, can be challenging and it can therefore be difficult to accurately measure the impact of interventions and other internal and external factors on financial security and those effects over time.

Obtaining validated income and benefits data, as a measure of financial security, could be a useful method of overcoming some of the subjectivity and challenges inherent with other self-reported measures. Validated income and benefits data can be obtained through financial statements or payslips. It may also be obtained through data linkage processes, acquiring validated income and benefits data from third party sources, such as the Department for Work and Pensions (DWP) and His Majesty’s Revenue and Customs (HMRC). The DWP is responsible for welfare, pensions and child maintenance policy in the UK [1]. They collect and hold personal and household data on benefits claimed and data on eligibility for benefits. HMRC is the tax, payments and customs authority of the UK government. It is responsible for collecting taxes, paying child benefits, enforcing tax and customs laws, and enforcing the payment of minimum wage by employers [2].

### Data linkage in health research

Data linkage processes can create rich data sets that provide a detailed picture of individuals and their families, communities and populations and can provide an effective way of obtaining objectively measured outcome variables [3].. Such data can permit entire populations to be studied and reduces common follow-up problems encountered in survey-based research designs. Data linkage processes can be seen as less intrusive and costly than collecting bespoke data, and allows entire populations to be studied over longer periods of time [4]. Data sets created through data linkage processes are, however, limited by the quality and completeness of the original data sources and researchers have little ability to influence the data or quality of data collected from the various sources. The quality of the resulting data is influenced by linkage error which is known to bias particular population groups, such as individuals from ethnic minority groups. Furthermore, Individuals and groups working within the grey economy, an informal economy that is neither taxed nor monitored by any form of government is unlikely to be captured within data linkage databases with the same levels of accuracy and completeness as other individuals.

Although health data is regarded as personal and private, it appears acceptable to share in a trusted medical context. Research exploring the perceptions of people from ethnic minority communities have found that they are more concerned about health data linkage than other groups [5]. However, they have also described how they often do not see evidence of data on their race, ethnicity, culture, or religion often being considered and feel under-represented in research [6]. Data linkage for health and educational records in the Born in Bradford Better Start (BiBBS) cohort study is reported to be high; greater than 99% of participants consent to data linkage with health and educational records [7].

### Financial data linkage in health research

There are a number of longitudinal population studies conducting health research that have already established data linkage pathways with the DWP and HMRC in order to better understand the impact of their research on the financial security of their participants [8, 9]. The English Longitudinal Study of Aging (ELSA) reports a 79% consent rate for linkage of participants to their benefits and income data through their national insurance number [10]. The Next Steps Age 25 Survey reports consent rates of 70% for data linkage with DWP and 65% for data linkage with HMRC [11].For the Avon Longitudinal Study of Parents and Children (ALSPAC) and Project to Enhance ALSPAC through Record Linkage (PEARL) studies, the consent rates for data linkage are slightly lower at 59% for the DWP and 57% for HMRC [8, 12]. Overall, the level of consent for data linkage in such longitudinal population studies remains higher for other records, such as educational and health records, than for financial records. Where done well data linkage can ameliorate research inequities through improvements in data completeness and representativeness. However, without careful design and implementation, information governance barriers and the potential for bias can risk exacerbating inequalities in longitudinal population studies utilising data linkage.

### Research aims

This research aims to explore attitudes and variation by sociodemographic factors in consent provided for financial data linkage in an experimental birth cohort study, in order to obtain validated income and benefits data, to better understand the impact on community interventions on the financial security of its participants and their families.

## Methods

### Study design

This research utilises an observational study design to explore consent rates, attitudes and variation in sociodemographic factors between participants of an experimental birth cohort who consent and do not consent to financial data linkage.

### Data collection

Born in Bradford (BiB) research programme is an internationally recognised, applied health research programme comprising health and wellbeing information on more than 30,000 Bradfordians enrolled in a family of three large, multi-ethnic prospective birth cohort studies: BiB Family; BiBBS; and BiB4All [13]. The BiB cohort studies comprises three discrete cohort populations and utilise different research designs.

BiBBS is a Big Lottery funded innovative experimental birth cohort, established in 2016, that simultaneously evaluates the impact of multiple early life interventions to improve outcomes for pregnant women and families with children aged 0–3 years in three inner city deprived, multi-ethnic wards in Bradford. It specifically explores: social and emotional development; communication and language development; and nutrition and obesity [14].

**Fig. 1:**
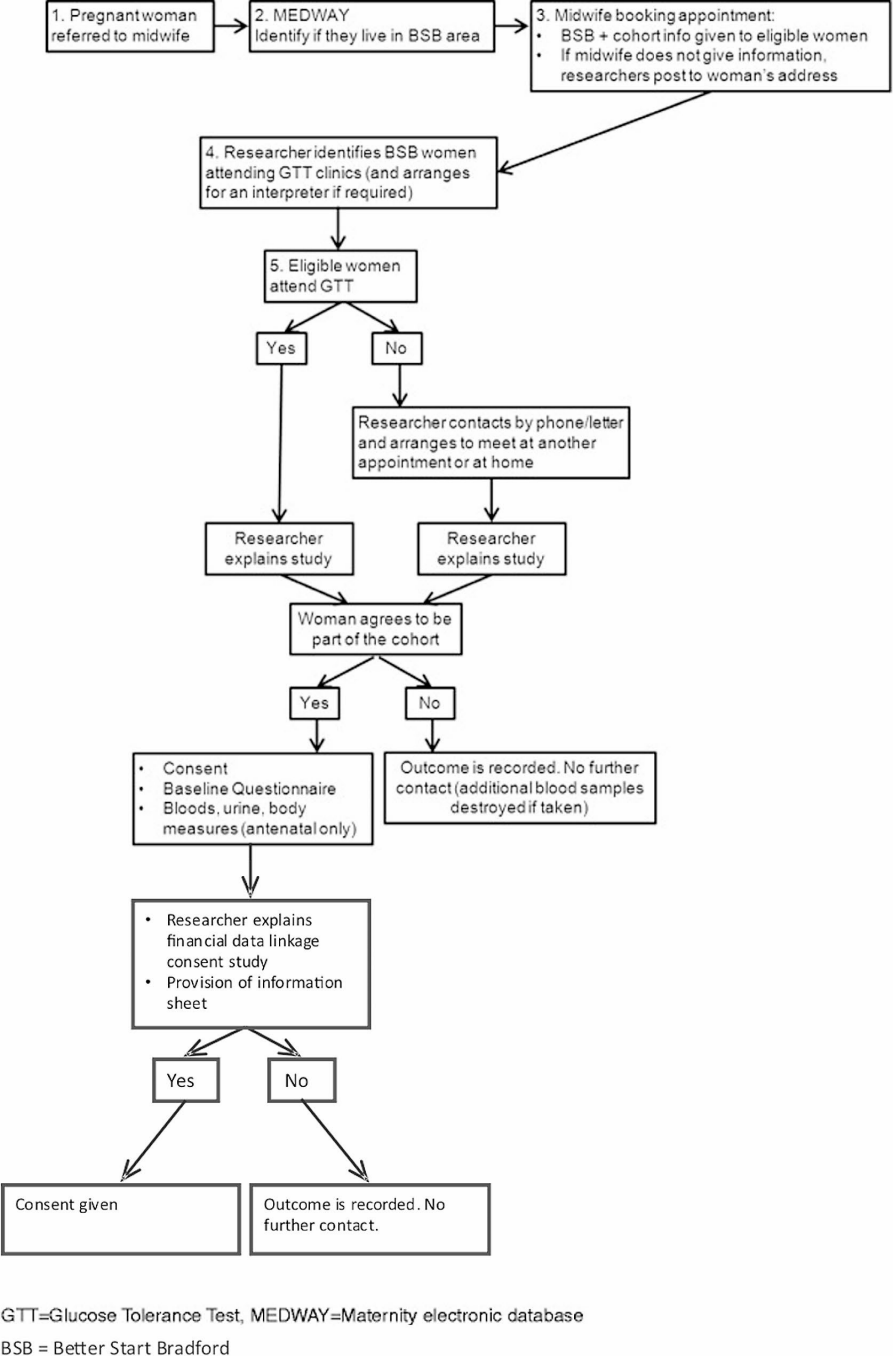
Recruitment process

Women are recruited from the Bradford Royal Infirmary maternity unit as they attend the clinic for an oral glucose tolerance test, routinely offered to all pregnant women, see Fig. 1. Recruitment is conducted face-to-face by the BiBBS recruitment team. The BiBBS recruitment protocol is described in full elsewhere [14]. Recruitment into the BiBBS cohort is ongoing and, as of the end of August 2023, 5000 women and their children are in the cohort [14]. BiBBS test interventions utilising trials within cohorts and other quasi-experimental designs, where trials within cohorts are neither feasible nor ethical, to evaluate early life interventions.

Participants in the BiBBS cohort are asked to provide consent for routine data linkage to health and education data for research. Participants in the BiB Family Cohort Study and BiBBS cohort study also complete detailed questionnaires at recruitment and in ongoing waves of data collection, including information regarding wider social determinants of health ^61^. Questionnaires do not collect detailed financial data regarding income or benefits claims and entitlement because when these data items were piloted, there were high levels of missingness either because women did not know this information or did not wish to answer financial questions. Additional data linkage pathways have recently been established between the BiBBS cohort and the DWP and HMRC and a trial of obtaining consent for financial data linkage has been trialled within the cohort.

Immediately following recruitment to the BiBBS cohort study, participants were asked by the BiBBS recruitment team whether they would also be willing to consent to further data linkage with the DWP and HMRC, as an addendum to the usual BiBBS recruitment process. Participants were provided with a participant information sheet and separate consent form to provide their consent to financial data linkage, see Additional File 1. Data collection ran from the 1st October 2021 to 27th February 2023.

### Outcome measures

Participants were coded as having provided consent or having declined to consent to data linkage with the DWP and HMRC. Participants who did not wish to provide consent for financial data linkage were asked for their reason for not wishing to providing consent. Their response was entered as a free text response. Where it was not feasible for recruitment coordinators to obtain consent, for example because a participant became unwell during the recruitment process or lack of administrative time, participants were coded as having declined consent and a reason was documented to explain the reason why.

Participant sociodemographic data was taken from the BiBBS baseline cohort study questionnaires [14]. The key domains used in this analysis were: ethnicity; socioeconomic security; language; relationships; and health and wellbeing.

Ethnicity was coded using Census 2011 categories as ‘White British’, ‘Pakistani Heritage’ and ‘Other’. There were small numbers of non-White British and non-Pakistani Heritage mothers from multiple ethnic groups (White Polish/ Slovakian/Romanian/ Czech, Other White, British Indian/Indian, British Bangladeshi/Bangladeshi, Black Caribbean, Black African, Mixed heritage, Other, Do not wish to answer) who were grouped and categorised within the ‘Other’ category.

To establish financial insecurity, the surveys employed the question: ‘How well would you say you are managing financially right now?’. Answer options included: ‘living comfortably’; ‘doing alright’; ‘just about getting by’; ‘finding it quite difficult’; and ‘finding it very difficult’. The latter two options were grouped and categorised as indicating financial insecurity. Participants were also asked: ‘Compared to a year ago, how would you say you (and your partner) are doing financially?’. Answer options included: ‘better off’; ‘about the same’; and ‘worse off’. Residential address, as at 31st March 2019, was linked to the 2019 Index of Multiple Deprivation [15]. Participants were also asked their employment status and the employment status of their partner, where relevant.

The relationship status of the participants was recorded and whether the baby’s father lived with the mother. If a partner, friend or relative was present with the mother at the time of the survey, this was also coded.

Language was coded as ‘English’, ‘Punjabi’, ‘Urdu’, ‘Pashto’, ‘Bengali’ or ‘Other’. There were small numbers of non-British, Punjabi, or Urdu speaking mothers who were grouped and categorised within the ‘Other’ category. First language was further grouped into ‘English’ and ‘Language other than English’. Where English was not the first language, participants were asked how well they could read, write and speak English. Answer options included: ‘not at all’; ‘a little bit’; ‘some’; ‘quite well’; and ‘very well’. The latter two options were grouped and categorised as indicating comprehension of the English language for each domain.

The general health and wellbeing of participants was measured using several tools. Participants were asked for a self-reported measure of their general health: ‘How would you describe your health generally?’. Answer options included: ‘Excellent’; ‘Very good’; ‘Good’; ‘Fair’; and ‘Poor’. The first three options were grouped and categorised as good health, with the latter two options being grouped as indicating poor self-reported general health.

Mental health, wellbeing and health-related quality of life were measured using the PHQ-8, SWEMWBS and EuroQol EQ-5D tools respectively [16–18]. Mental health was measured using the PHQ-8 questionnaire [16]. The scores from each item were summed to produce a total score between 0 and 24 points. Summed scores were used as a continuous variable with greater scores indicating a presence of depressive symptoms. Standard categorisations were employed for the scores: 0 to 4 no depression; 5 to 9 mild depression; 10–14 moderate depression; and 15–24 severe depression [19]. Symptoms suggestive of depression were defined as those with moderate to severe depression scores.

Wellbeing was measured using the seven-item SWEMWBS [20]. The score from each item was summed to produce a total score between 14 and 35. Summed scores were transformed and used as a continuous variable with greater scores indicating a more positive wellbeing. SWEMWBS scores were further categorised into low (7-19.5), average (19.6–27.4) and high (27.5–35) wellbeing groups.

The health-related quality of life of participants was measured using the five-item EQ-5D instrument (EQ-5D-5 L) [21]. These domains provide a descriptive profile that were transformed into health utility scores, based on UK societal preference weights for the health state, [22] ranging between 0 representing death and 1 for perfect health. The EQ-5D-5 L questionnaire also includes a Visual Analog Scale, by which respondents can self-report their perceived health status with a continuous grade ranging from 0 representing the worst possible health to 100 representing the best possible health.

### Sample size

It was calculated that 519 participants would be required to have 90% power to detect a consent rate for financial data linkage comparable to that reported by other longitudinal studies of 60%, for a 5% two-sided alpha t-test [10–12, 23, 24].

### Data analysis

Descriptive statistics were used to identify the overall proportion of BiBBS participants who consented to data linkage with the DWP and HMRC and their sociodemographic characteristics are reported. Multiple logistic regression models were used to explore associations between provision of consent and key explanatory variables, including: ethnicity; socioeconomic security; language; relationships; and health and wellbeing. Missing data on measures was small for most variables and was not adjusted for in the analyses. All statistical analyses were carried out using Stata 15 [25].

Free text responses explaining the reason for declining consent were coded and analysed using a thematic analysis to explore reasons participants did not wish to provide consent for data linkage with the DWP and HMRC [26]. Qualitative analysis was conducted using NVivo10 [27].

### Patient and public involvement and Engagement

BiBBS is supported by the Born in Bradford Community Research Advisory Group (CRAG). The CRAG comprises community representatives, including engagement workers, local parents, leaders of local groups, projects and charities and local councillors. The CRAG was involved in the co-production of the BiBBS cohort study and continues to work in partnership with BiBBS by helping to engage with the local community and provide feedback on successes and challenges [14]. The CRAG and research team worked with representatives from the DWP to establish a data sharing pathway between the BiB Research Programme and the DWP to facilitate data linkage between BiBBS participants and their income and benefits data, held by the DWP and HMRC.

To permit financial data linkage, a participant information sheet and consent form was developed, in consultation with the BiBBS research and recruitment teams and the CRAG. Initial models proposed to obtain consent for financial data linkage included: (a) Obtaining consent for financial data linkage following standard recruitment into the BiBBS cohort, as a distinct and discrete consent process (b) Obtaining consent for financial data linkage as part of the usual recruitment into the BiBBS cohort. Participants would be asked if they consent to financial data linkage alongside their educational and health records, as part of one unified consent process (c) An opt-out approach to obtaining consent for all data linkage.

A model of consent was developed from existing, approved models of consent used by the ELSA study with established data linkage pathways with the DWP and HMRC, obtaining consent for financial data linkage following standard recruitment into the BiBBS cohort [28]. This model was considered the most transparent, which was important for the patient and public representatives, and which already met governmental and legal requirements for financial data linkage.

## Results

### Study population

**Fig. 2:**
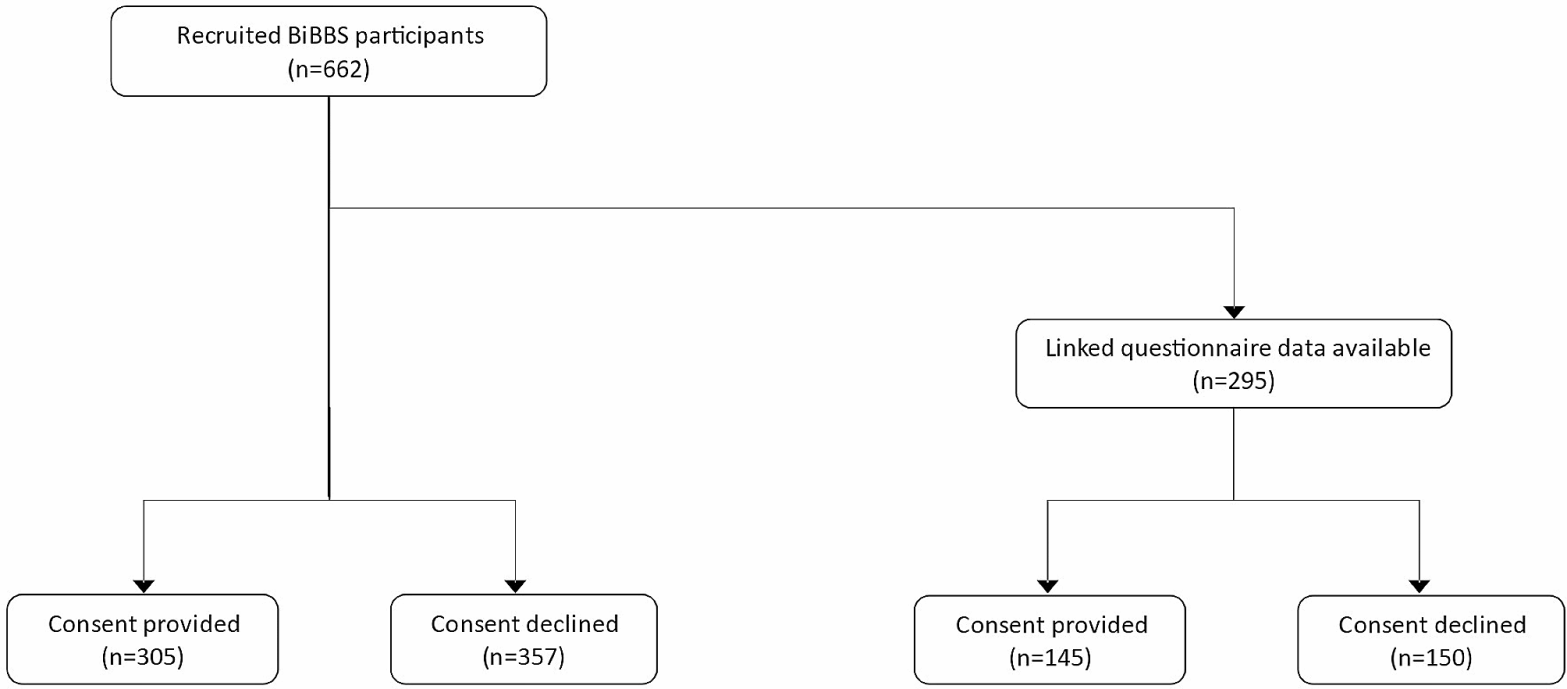
Consort diagram

The pilot study ran from the 1st October 2021 to 27th February 2023. A total of 662 participants were recruited into the BiBBS study and were asked if they were willing to provide consent for financial data linkage, see Fig. 2. As BiBBS is an ongoing cohort, an interim data extraction was completed for the purposes of this analysis. As a result, only questionnaires which were completed face-to-face during the pilot study were available, providing linked sociodemographic data for 295 participants, see Table 1.

**Table 1:** Sample characteristics of the study population with available linked sociodemographic data

	Number (*n* = 295)	Frequency (%) (95% CI)
**Ethnicity**		
White British	25	8 (6, 12)
Pakistani	177	60 (54, 65)
Other	93	32 (26, 37)
Missing	0	
**IMD 2019 decile**		
IMD 1 (most deprived)	91	81 (73, 88)
IMD 2	21	19 (12, 27)
IMD 3–10 (least deprived)	0	0 (0, 0)
Missing	183	
**Employment status**		
Employed	183	62 (56, 67)
Not employed	112	38 (33, 44)
Missing	0	
**Financial security**		
Living comfortably	72	26 (21, 31)
Doing alright	104	37 (32, 43)
Just about getting by	71	25 (21, 31)
Finding it quite difficult	22	8 (5, 12)
Finding it very difficult	11	4 (2, 7)
Missing	15	
**Relationship status**		
Married	242	82 (77, 86)
In a relationship but not married	32	11 (8, 15)
Separated or divorced	15	5 (3, 8)
Never been in a relationship with father	6	2 (3, 8)
Partner has died	0	0 (0, 0)
Missing	0	
**Single parent**		
Yes	42	14 (11, 19)
No	253	86 (81, 89)
Missing	0	
**Partner employment status**		
Employed	228	62 (56, 67)
Not employed	37	38 (33, 44)
Missing	30	
**Person present at recruitment**		
None	171	58 52, 65)
Partner	60	3 (2, 6)
Family member	9	1 (0, 3)
Friend	< 5	4 (3, 7)
Other	< 5	
Missing	< 5	
**First language**		
English	109	37 (32, 43)
Punjabi	79	27 (22, 32)
Urdu	35	12 (9, 16)
Pashto	15	5 (3, 8)
Bengali	21	7 (5, 11)
Other	36	12 (9, 16)
Missing	0	
**Where English is not first language: how well can understand verbal communication in English**		
Not at all	< 5	2 (1, 6)
A little bit	26	14 (10, 20)
Some	22	12 (8, 17)
Quite well	49	26 (20, 33)
Very	85	46 (39, 53)
Missing	9	
**Self-reported general health**		
Excellent	15	5 (3, 8)
Very good	57	20 (15, 25)
Good	140	48 (43, 54)
Fair	62	21 (17, 26)
Poor	16	6 (3, 9)
Missing	5	
**PHQ-8 score**		
PHQ-8 mean score	13.8 (95% CI 13.3, 14.4)	
**PHQ-8 Category**		
No depression	90	34 (29, 40)
Mild depression	133	50 (44, 56)
Moderate depression	41	16 (12, 20)
Severe depression	0	0 (0, 0)
Missing	31	
**Clinically relevant symptoms of depression**		
No clinically relevant symptoms of depression	223	84 (80, 88)
Clinically relevant symptoms of depression	41	16 (12, 20)
Missing	31	
**SWEMWBS score**		
SWEMWBS mean score	26.7 (95% CI 26.0, 27.4)	
**SWEMWBS Category**		
Low wellbeing	33	11 (8, 16)
Moderate wellbeing	125	44 (38, 49)
High wellbeing	129	45 (39, 510
Missing	8	
**EQ-5D utility score**		
EQ-5D mean utility score	0.117 (95% CI 0.99, 0.135)	

Participants with linked sociodemographic data were ethnically diverse: 177 (60%) were of Pakistani heritage; 25 (8%) were White British; and 93 (32%) comprised of mothers from other ethnic groups. The majority of participants lived in the first (91%) most deprived IMD decile in England, with the remainder living in the second (22%) most deprived IMD decile. Most participants were employed (62%). Participants were more likely to report that they were living comfortably (26%), doing alright (37%) or just about getting by (25%). Some participants reported that they were finding it quite (8%) or very difficult (4%) to manage financially.

Most participants (82%) reported that they were married or in a relationship with the father of their child but not married (11%). A small proportion of participants reported that they separated or divorced (5%) or have never been in a relationship with the father (2%). 42 (14%) mothers were single parents. Where relevant, most participants reported that their partners were employed (62%).

The first language reported by participants varied across the study population: 109 (37%) reported English was their first language; 79 (27%) Punjabi; 35 (12%) Urdu; 21 (7%) Bengali; 15 (5%) Pashto; and 36 (12%) participants reported having ‘Other’ first language. Where English was not a participant’s first language, most participants reported that they could understand spoken English very well (46%) or quite well (26%).

Participant’s self-reported health varied across the study population. The majority of participants felt that they had good health (48%). Similar proportions of participants felt that their general health was very good (20%) and fair (21%). Overall across the study population, the mean PHQ-8 score was 13.8 (95% CI 13.3, 14.4) with 50% of participants falling within the mild depression category and 34% of participants falling within the no depression category. The majority of participants had moderate (44%) to high (45%) wellbeing scores. The mean SWEMWBS score for the study population was 26.7 (95% CI 26.0, 27.4) falling towards the higher end of the moderate wellbeing category. The mean EQ-5D utility score for the study population was 0.117 (95% CI 0.99, 0.135). Participants with linked sociodemographic data were representative of the BiBBS cohort and Bradford population with respect to sample characteristics [7, 29].

### Consent rates for financial data linkage

Consent rates for financial data linkage are reported overall for the study population and separately for participants with linked sociodemographic data available, see Table 2. Of the 662 participants, 46% (95% CI 41%, 50%) provided consent for financial data linkage and 54% (95% CI 49%, 58%) declined consent for financial data linkage. Overall, participants were less likely to provide consent after controlling for ethnicity spoken, language spoken, financial security and employment status (aOR 0.98 95% CI 0.82, 1.34). For participants where linked sociodemographic data was available, 49% (95% CI 43%, 55%) provided consent for financial data linkage and 51% (95% CI 45%, 57%) declined consent for financial data linkage.

**Table 2:** Consent rates for financial data linkage for all participants and participants with linked sociodemographic data

	All participants		Participants with linked sociodemographic data	
	Number	Frequency (%) (95% CI)	Number	Frequency (%) (95% CI)
Provided consent	305	46 (41, 50)	145	49 (43, 55)
Declined consent	357	54 (49, 58)	150	51 (45, 57)
Total	662	100	295	100

Overall, participants were equally likely to decline consent to financial data linkage. Given the overlap of confidence intervals between each consent group, the difference is not statistically significant.

### Variation in consent rates for financial data linkage

Multiple logistic regression models were used to explore associations between provision of consent and key explanatory variables, including: ethnicity; socioeconomic security; language; relationships; and health and wellbeing, see Table 3. Measures of socioeconomic insecurity were associated with being more likely to provide consent for financial data linkage. Participants who were not employed were more likely to provide consent for financial data linkage (OR 1.49 95% CI 0.93, 2.40), although this was not statistically significant. Participants who were more financially insecure were more likely to provide consent for data linkage than those who reported that they were living comfortably. Participants who were finding it quite difficult to get by were most likely to provide consent for data linkage than those who reported that they were living comfortably (OR 4.30 95% CI 1.54, 11.91). Overall, participants who were more financially insecure were more likely to provide consent than those who were financially secure (OR 1.85 95% CI 1.14, 3.93).

**Table 3:** Odds ratios (95% CI) from unadjusted logistic regression model for the probability of providing consent

	OR	95% CI
**Ethnicity (Reference: White British)**		
Pakistani	0.76	0.33–1.76
Other	1.17	0.48–2.84
**Employment status (Reference: Employed)**		
Not employed	1.49	0.93–2.40
**Financial security (Reference: Living comfortably)**		
Doing alright	2.43	1.30–4.53
Just about getting by	2.44	1.24–4.80
Finding it quite difficult	4.30	1.54–11.91
Finding it very difficult	2.40	0.66–8.67
**Financial security (Reference: Financially secure)**		
Financial insecurity	1.85	1.14–3.93
**Relationship status (Reference: Married)**		
In a relationship but not married	1.40	0.66–2.93
Separated or divorced	2.17	0.72–6.54
Never been in a relationship with father	0.22	0.03–1.89
**Single parent (Reference: Not single parent)**		
Single parent	1.16	0.60–2.24
**Partner employment status (Reference: Employed)**		
Not employed	1.86	0.91–3.79
**Person present at recruitment (Reference: None)**		
Partner, family or friend	0.73	0.45–0.96
**First language (Reference: English)**		
Punjabi	0.59	0.33–0.98
Urdu	0.46	0.21–0.94
Pashto	0.39	0.13–1.23
Bengali	1.05	0.41–2.69
Other	0.98	0.46–2.10
**First language (Reference: English)**		
Language other than English	0.65	0.39–0.98
**Where English is not first language: how well can understand verbal communication in English (Reference: Not well)**		
Well	0.85	0.48–1.52
**Self-reported general health (Reference: Good health)**		
Poor health	1.31	0.78–2.20
PHQ-8 score		
PHQ-8 mean score	1.00	0.95–1.06
**PHQ-8 category (Reference: Mild depression)**		
Moderate depression	0.97	0.48–1.95
Severe depression	1.04	0.50–2.18
**Clinically relevant symptoms of depression (Not clinically depressed)**		
Clinically relevant symptoms of depression	1.00	0.51–1.94
SWEMWBS score		
SWEMWBS mean score	0.98	0.94–1.02
**SWEMWBS Category (Reference: Low wellbeing)**		
Moderate wellbeing	1.09	0.50–2.34
High wellbeing	0.75	0.35–1.60
**EQ-5D utility score**		
EQ-5D mean utility score	1.00	1.00–1.00

Relationship factors such as relationship status and whether the participant is a single parent were not associated with whether a person is likely to provide consent for financial data linkage. Similarly, the employment status of the participant’s partner and whether and the type of person present at recruitment were not associated with the provision of consent.

Ethnicity was not a factor that was associated with consent for data linkage. However, there were several indicators that factors relating to language spoken and the comprehension of the English language were associated with the probability of providing consent for financial data linkage. Overall, where the participant’s first language was a language other than English, participants were less likely to provide consent for data linkage (OR 0.65 95% CI 0.39, 0.98). At the level of individual languages, this association persisted and was significant for participants who spoke Punjabi (OR 0.59 95% CI 0.33, 0.98) and Urdu (OR 0.46 95% CI 0.21, 0.94) compared to participants who spoke English as their first language. This association was not significant for those who spoke Pashto (OR 0.39 95% CI 0.13, 1.23), Bengali (OR 1.05 95% CI 0.41, 2.69) or another language (OR 0.98 95% CI 0.46, 2.10) as their first language, which is likely to be related to the smaller participant numbers in these groups. However, where English was not the participants first language, how well a participant could understand verbal communication in English (OR 0.85 95% CI 0.48, 1.52) was not associated with whether a participant was likely to provide consent for financial data linkage.

There was no association found between measures of general health, mental health, wellbeing and health-related quality of life and the provision of consent for financial data linkage.

### Attitudes towards consent

All participants (*n* = 357) who declined to consent to financial data linkage were asked why they chose not to provide consent for data linkage. Of participants who declined to consent to financial data linkage, a reason for choosing to decline consent was not documented for 21 (6%) of participants. Some participants gave more than one reason (*n* = 132). The explanations for choosing not to give consent were coded, analysed and are documented in Table 4.

**Table 4:** Reasons for choosing to decline to consent to financial data linkage given by BiBBS participants

Reason given	Number	Frequency (%)
Not interested in taking part	139	39%
Does not feel comfortable sharing this sort of information	68	19%
Need to seek permission from partner prior to providing consent	24	7%
Partner deals with finances	24	7%
Participant is not in receipt of benefits and therefore did not understand the need to share financial data	23	6%
Partner, friend or other family member present during recruitment process and declined consent	12	6%
Did not understand the need to share financial data	19	5%
Participant does not understand finances	8	2%
Participant is already in receipt of benefits and therefore did not understand the need to share financial data	6	2%

Most participants explained that this aspect to the research programme was not something that they were interested in taking part in (39%). Some participants explained that they did not feel comfortable sharing this sort of information given the nature of the information (19%).

I don’t like it.

Participant.

Many reasons given by participants for not wishing to consent to financial data linkage related to a lack of understanding of the need to do so. In some cases this was stated as the reason with no further explanation given (5%). Some participants were already in receipt of benefits and felt they were already aware of their entitlement and therefore could not see how they would personally benefit from sharing such data (2%). Conversely, some participants who were employed and were not in receipt of benefits did not understand the need to share financial data (6%).

Husband deals with it and he’s working full time so might not get anything.

Participant.

Several participants explained that they did not understand finances or their financial circumstances (2%) or did not manage household finances (7%) and therefore felt unable to discuss any aspects of financial data sharing.

[my] husband deals with all that. [I don’t] know anything.

Participant.

Other participants stated that they would be unable to provide consent without the permission of their partner (7%). On occasion, the participant’s partner, friend or another family member (6%) were present during the recruitment process and decline to consent on behalf of the participant.

[I] need to ask [my] husband…can’t decide on [my] own.

Participant.

In some circumstances, recruitment coordinators were unable to seek consent from participants for financial data linkage. These reasons were coded, analysed and are documented in Table 5.

**Table 5:** Reasons documented by recruitment coordinators for being unable to request consent for financial data linkage

Reason documented	Number	Frequency (%)
Only recently moved to UK	10	3%
No national insurance number	9	3%
Other (including language barrier, immigration status, lack of time, illness)	< 5	< 2%

Some of the reasons documented by recruitment coordinators for not being able to seek consent for financial data linkage involved the participant not having lived in the country long (3%), not having a national insurance number (3%) and being a refugee or asylum seeker (< 5%).

Recently moved to UK. Not familiar with culture.

Recruitment coordinator.

Some recruitment coordinators documented language barriers (< 5%) as being a reason for being unable to seek consent, despite the presence of translators in many circumstances. Being physically located in a health setting, participants were sometimes called for healthcare appointments (< 5%) or became unwell (< 5%) before or during the process of seeking consent.

## Discussion

### Summary of key findings

Overall, participants were equally likely to consent and decline consent for financial data linkage. There were some factors relating to socioeconomic security and language spoken found to be associated with the decision to consent to financial data linkage. Measures of socioeconomic insecurity were associated with being more likely to provide consent for financial data linkage. Participants who were not employed (OR 1.49 95% CI 0.93, 2.40) and were more financially insecure (OR 1.85 95% CI 1.14, 3.93) were more likely to provide consent for financial data linkage.

However, there were several indicators that language spoken was associated with the probability of providing consent for financial data linkage. Overall, where the participant’s first language was a language other than English, participants were less likely to provide consent for data linkage (OR 0.65 95% CI 0.39, 0.98). However, where English was not the participants first language, how well a participant reported that they could understand English was not associated with whether a participant was likely to provide consent for financial data linkage. Ethnicity was also not found to be a factor that was associated with consent for financial data linkage.

Furthermore, the choice of consent for financial data linkage was not associated with: relationship factors, such as relationship status and whether the participant is a single; employment status of the participant’s partner; person present at time of recruitment; and measures of health, such as general health, mental health, wellbeing and health-related quality of life.

For participants who chose not to provide consent for financial data linkage, a number of varied explanatory reasons were provided. Most participants reported feeling that they were simply not interested in taking part in research involving financial data sharing and did not feel comfortable sharing such sensitive data. Some participants highlighted that they did not understand the need for sharing financial data and related this to being financially secure or already being in receipt of benefits. In some circumstances, participants felt that because they did not understand their finances or because their partner organised the household financial circumstances, they felt unable to participate or to provide consent to participate and would need their partner’s permission to do so. On some occasions, a partner or family member was also present during the recruitment process and expressed concerns about financial data linkage. For this reason the participant declined to consent to data linkage with their financial records.

In some circumstances, recruitment coordinators were unable to seek consent from participants for financial data linkage. Commonly, this related to language barriers, or the participant becoming unwell or needing to go to a health appointment prior to or during the process of obtaining consent. Sometimes recruitment coordinators documented that they were unable to seek consent because the participant had not been in the country long or did not have a national insurance number.

### Limitations

The pilot of the chosen approach to obtaining consent for financial data linkage was subject to a number of limitations. Nearly half of participants did not have linked sociodemographic data available. This was related to delays in the processing of BiBBS recruitment data and should be available at a later date. However, the omission of these participants in the analysis, may have implications on the analysis and subsequent interpretation of the results.

A number of sociodemographic variables were collapsed to support the analysis owing to small sample sizes within each strata across survey timepoints. For example, financial insecurity was defined as those ‘finding it quite difficult’ and ‘finding it very difficult’ to manage financially. Families ‘living comfortably’, ‘doing alright’ and ‘just about getting by’ were considered financially secure. Such categorisations are conservative and several mid-point categories could be considered true for either categorisation. This is likely to have underestimated the measure of association for these groups.

Finally, the reasons documented for declining consent were often simple and the wording similar between participants. This perhaps reflected a lack of administrative time and the confidence and understanding of the recruitment coordinators with respect to seeking consent for data linkage and discussing sensitive information. Furthermore, it was unclear to what degree the reason for declining consent documented was in the participant’s own words and the degree to which the reason given was understood and interpreted by the recruitment coordinators prior to documentation. This highlights the need to understand the reflexivity of the recruitment coordinators involved to better understand the effect of this on the results and the possible introduction and extent of bias involved.

### Implication of findings

Financial information is widely regarded as sensitive data and being financially insecure and being in receipt of benefits can be stigmatising. This can lead to challenges in discussing financial circumstances for the purposes of research and may mean financial insecurities are under or overestimated. For some financial circumstances are simply not known or fully understood and is an area that could be explored further in this particular cohort to better understand and measure this area. This research highlights the potential for the use of data linkage as a means to obtain objective, validated financial data to improve the accuracy and understanding of financial circumstances and insecurity in research. However, the utility of such financial data as objective and validated measures of financial circumstances as used in health research are not yet fully understood and requires further research. It is understood that such data will provide clarity of income and receipt of benefits, which may give some indication to benefit entitlement, uptake of advice for those who are eligible and outcomes for those who access intervention to improve financial security. However, the information obtained may not be sufficient to capture all individuals who are eligible and who may not claim benefits. Furthermore, data linkage in longitudinal population based studies based on a consent rate of 46% could introduce significant biases. The lack of this information may disproportionally impact vulnerable groups who are likely to have disengaged with the benefits system, such as homeless people or refugees, and still not have found work or be consistently in work. For longitudinal population studies incorporating financial data linkage it is imperative that efforts are made to develop data linkage processes, including consent methodology, in genuine collaboration with the population that the studies represent to improve engagement and representativeness of those who consent. Further research is required to better understand how alternative forms of communication and method of consent affect engagement in this population. Furthermore, the definition of financial security may need to be re-examined and not taken in totality to represent household income and the presence or absence of debt. Measures of financial resilience need to be captured and further reinforces the need to supplement any objective data received with important subjective self-reported measures of financial security. This reflects the need to conduct further research into how such subjective measures reflect the experience of financial resilience and security for individuals and their families.

Research has highlighted that some data uses and data linkage processes are more concerning for participants than others and that context is important. People trust the health sector, including health researchers, more than they trust other organisations, such as the DWP and HMRC [30, 31]. Research also highlights that trust in the health sector could be at risk of being diminished by the involvement of less trusted parties [5]. It is also important to understand that the potential for financial data linkage in a study may affect the type of person willing to participate in a study and may exclude those most vulnerable to financial insecurity. Furthermore, the requirements for financial data linkage with governmental agencies are stringent, making the consent process seem more daunting for researchers and participants alike. It highlights the need to investigate this process further to test acceptability of asking for consent to this data linkage given the impact this could have on participant recruitment, causing cohorts to become unrepresentative and damaging trust within communities.

This research improves our understanding of the impact of the use of financial data on recruitment rates for cohort studies used in health research. The consent rates found in this study were lower for this study (46%) than for other published consent rates for other longitudinal population studies of 79% for the ELSA study, of 70% (DWP) and 65% (HMRC) for the Next Steps Age 25 Survey and 59% (DWP) and 57% (HMRC) for the ALSPAC and PEARL studies [11, 12]. This may reflect differences in consent methodology between the studies. Some interstudy variation was reported for the Next Steps, ALSPAC and PEARL studies with respect to method of communication when obtaining consent, with face-to-face and telephone communication achieving higher rates if consent than online methods [11, 12]. This highlights the complex nature of utilising data linkage in health research and the interplay of consent and communication methodology with this. Furthermore, the differences in consent rates between these studies may reflect inherent differences within the population group. The BiBBS population group represent a young and pregnant population in an ethnically diverse and deprived population.

This research further improves our understanding of the interplay of socioeconomic demographics on the provision of consent for financial data linkage. It suggests that adapting consent procedures in light of these findings may improve rates of consent, in particular for the most vulnerable and marginalised groups. However, further entwining of financial data linkage consent pathways into the existing BiBBS recruitment process may impact upon the relationship and trust the research group have built with the participants and its community, which has been reflected in other health research studies. Therefore ongoing careful and meaningful co-production of consent processes with patients and the public, representative of this cohort is imperative. Further research needs to be conducted with larger sample sizes to fully understand some of the reported associations, particularly with certain marginalised groups, where sample sizes are inherently smaller. Particularly, there needs to be more research conducted specifically to understand how attitudes towards financial data sharing relates to language spoken and how best to overcome this, particularly with respect to how consent is obtained for financial data linkage. However, the research suggests that those experiencing financial insecurity overall are more likely to consent to data linkage and as such, it is possible that data linkage would be achieved for those most likely to need welfare advice.

## Conclusion

Improving our understanding of what constitutes financial insecurity can help us to better understand how financial insecurity is experienced and how this can change over time within and between individuals and populations. Having an accurate understanding of financial insecurity can also help researchers, commissioners and policy makers to have a better understanding of what works to improve the financial security of individuals and communities and the impact this has on health and wellbeing and health insecurity.

Any approach to obtaining financial data from participants needs to be sensitive and considered. It should be acceptable to all agents involved and meeting ethical requirements of governmental and research institutions. Pragmatic, imaginative and flexible approaches are needed if research using data linkage is to successfully realise its potential for public good without undermining trust in the research process.

This research sets out an approach to obtaining validated income and benefits data, as a proxy measure for financial security, within the particular context of the BiBBS cohort study. It achieves good consent rates and demonstrates greater input from those who report greater potential need for financial support. Further research should be conducted to further understand the interplay of language spoken in this context.

### Electronic supplementary material

Below is the link to the electronic supplementary material.

**Additional File 1**: “Information sheet to link your income and benefits data and consent form.” File contains participant information sheet and consent form used to obtain consent for financial data linkage

## Data Availability

This data is available through a system of managed Open Access. Researchers who would like access to this data, or any other Born in Bradford data are encouraged to submit an expression of interest which will be reviewed by the BiB Executive (who meet to review proposals on a monthly basis and will endeavour to respond to your request as soon as possible). If your request is approved we will ask you to sign a Data Sharing Contract and a Data Sharing Agreement. For further information please see: How to access data - Born In Bradford.

## Abbreviations

ALSPAC: Avon Longitudinal Study of Parents and Children
BiBBS: Born in Bradford’s Better Start
CI: Confidence interval
CRAG: Community Research Advisory Group
DWP: Department for Work and Pensions
ELSA: English Longitudinal Study of Aging
HMRC: His Majesty’s Revenue and Customs
NIHR: National Institute for Health and Care Research
OR: Odds ratio
PEARL: Project to Enhance ALSPAC through Record Linkage
PHQ-8: Patient Health Questionnaire
SWEMWBS: Shortened Warwick-Edinburgh Mental Wellbeing Scale
WEMWBS: Warwick-Edinburgh Mental Wellbeing Scale
