# Supplementary material for: Exploring attitudes and variation by sociodemographic factors in consent provided for financial data linkage in an experimental birth cohort study
Source: BMC Public Health. 2024 Mar 5;24:712. doi: 10.1186/s12889-024-18226-1 (PMC10916313; doi:10.1186/s12889-024-18226-1)
Supplement: Supplementary file 1 — Additional File 1: “Information sheet to link your income and benefits data and consent form.” File contains participant information sheet and consent form used to obtain consent for financial data linkage [file 12889_2024_18226_MOESM1_ESM.docx]

**Information sheet to link your income and benefits data**

We would like to get your permission to link to some information about your income and the benefits your family receive.

In our questionnaire we ask all mums about how their family is managing financially. We would like to ask more detailed questions about family income, but some women find it hard to answer these questions. Instead, we would like to get your permission to get this information directly from the organisations that hold this information, so that we can look at the impact of finances on the health and wellbeing of families. We are also aware that some families might not be claiming the benefits that they are entitled to and this information will help us identify where extra support is available.

**What am I being asked to give permission for?**

The Department for Work and Pensions (DWP) keeps records of everyone’s benefit claims and time spent on employment programmes. HM Revenue and Customs (HMRC) keeps records of your income and contributions to the National Insurance system. We would like to link this information to the information you give us as part of the BiBBS study.

**Do I have to agree to this part of the study?**

No. You can still be a part of the BiBBS study even if you don’t want us to collect this information from DWP and HMRC. It is entirely up to you. You can also change your mind at any time.

**What will happen if I agree to this?**

If you agree to share your information, we will give the DWP and HMRC your name, address, sex and date of birth. It would be helpful provide your National Insurance number with this information, but you do not have to do so.

They will use these details to identify the correct records it holds about you. This information will then be sent to BiBBS who will add this information to your study responses. Your personal information will be removed before any research is done.

The DWP and HMRC will not use the information we give them to do any checks on you. The DWP and HMRC will not store the name, address, sex, date of birth and national insurance information BiBBS gave to them. The data will not be used to work out whether any individual is claiming benefits they should not be and will not affect any current or future claims for benefits.

**Who can see my information?**

We need your permission for any information to be given to us. Like everything else you have told us, the information will be completely confidential and will be used for research purposes only. It will be used by researchers under restricted access arrangements which make sure that the information is used responsibly and safely. Names and addresses are never included in the results.

We would like to keep the data at Born in Bradford as long as we have live projects that use the data or there is the possibility of future projects that will use the data. We will ensure that your records are secure for as long as we continue this study.

**What if I change my mind?**

You can withdraw permission at any time for us to have your DWP and HMRC information. If you want to withdraw please contact our project office by phone (01274 383941) or email ([bibbs@bthft.nhs.uk](mailto:bibbs@bthft.nhs.uk)).

**Consent to link your income and benefits data**

Name of Parent (print)……………………………………………………………………………

| 1a  1b  1c  1d | I have understood the Information Sheet and Consent for Linking of Economic Records (Version 1.0 23.07.2021). I have had the opportunity to consider the information, ask questions and have had these answered satisfactorily.  I understand that all the information about me will be treated in strict confidence in accordance with the relevant UK data protection regulations and used responsibly and safely for research purposes only. It will not be possible for anyone outside of BiBBS to link my information to me or to my child.  I understand that my participation is voluntary and that I am free to withdraw at any time without giving any reason, without my medical care or legal rights being affected. I understand that I can withdraw from the study by contacting BiBBS staff using the contact details at the bottom of this form. If we become aware that you have become unable to consent in the future, you will not collect any further information.  I authorise the Department for Work and Pensions (DWP) and Her Majesty’s Revenue and Customs (HMRC) to disclose to BiBBS information about my income and benefits entitlements. This consent will remain valid until revoked by me in writing. | Yes    Yes  Yes    Yes |
| --- | --- | --- |

Signed by Parent ……………………………………………………… Date: …………………

**Researcher confirmation**

I confirm that I have explained the nature of the proposed research to the person(s) named on this form and have left a copy of the Information Sheet to link your income and benefits data dated 23.07.2021. and this consent form with them for future reference.

Name of researcher (print)…………………………………………………………………………

Signed by researcher …………………………………………………. Date: ……………………

**Interpreter confirmation**

I confirm that I have interpreted the Information Sheet for Linking of Economic Records dated 23.07.2021 and this consent form with them and that the participant understands.

Name of interpreter (print) …………………………………………………………………………

Signed by interpreter ……………………………………………………Date: .…………………

**BiBBS contact details**

If you would like any further information or want to withdraw your permission, please contact us at:

Born in Bradford’s Better Start (BiBBS), Bradford Institute for Health Research, Temple Bank House, Bradford Royal Infirmary, Duckworth Lane, Bradford BD9 6RJ.

Phone: 01274 383941 Email: bibbs@bthft.nhs.uk
